# Gut Microbial Choline TMA-Lyase CutC: From Metabolic Mechanism to a Novel Therapeutic Target for Diseases

**DOI:** 10.3390/nu18111659

**Published:** 2026-05-22

**Authors:** Na Zhang, Ying Wang, Gan Luo, Xiaoyan Gao

**Affiliations:** School of Chinese Materia Medica, Beijing University of Chinese Medicine, Beijing 102488, China; zhangna935094@bucm.edu.cn (N.Z.); wangy174@bucm.edu.cn (Y.W.); 201701032@bucm.edu.cn (G.L.)

**Keywords:** choline TMA-lyase, trimethylamine N-oxide, gut microbiota, dietary intervention, enzyme inhibitors, cardiometabolic diseases

## Abstract

In recent years, the pivotal role of the gut microbiota and its metabolites in host health and disease has garnered increasing attention. Dietary phosphatidylcholine and choline are metabolized by gut bacteria to generate trimethylamine (TMA). Upon entering the bloodstream, TMA is oxidized by host liver enzymes to trimethylamine N-oxide (TMAO), a known independent risk factor for various systemic diseases, including atherosclerosis, thrombosis, and chronic kidney disease. Within this complex “diet–gut–host” metabolic axis, the microbial choline TMA-lyase (CutC) acts as the key rate-limiting enzyme that catalyzes the cleavage of choline to produce TMA. This review systematically summarizes the discovery history, enzymatic structural characteristics, and catalytic mechanism of CutC, highlighting its potential as a microbial metabolic target for treating associated diseases. By specifically analyzing existing inhibitor strategies and interventions, this article emphasizes the extensive potential of specific targeting of the CutC enzyme in precisely regulating the functions of the microecology.

## 1. Introduction

The gut microbiota is a vital mediator connecting the host’s internal environment with the external world and is considered a metabolic “organ” capable of regulating host metabolism [1]. The human intestine harbors over 1000 microbial species, with a total cell count exceeding 10^14^, approximately 1.3 times the number of human cells, and a gene count 38 times that of the human genome [2]. Often termed the “second genome,” this microbiota possesses a rich array of metabolic enzymes that interact with exogenous substances, such as dietary components and drugs, thus modulating disease processes [3,4,5]. It has been shown to play a significant role in the pathogenesis of various human metabolic diseases [6,7]. Choline is an essential human nutrient, involved in neurotransmitter synthesis and lipid metabolism. However, excess dietary choline is metabolized by human gut anaerobic microorganisms as a carbon and energy source, converting it into trimethylamine (TMA), a specific microbial metabolite. TMA is subsequently oxidized in the host liver by flavin-containing monooxygenase 3 (FMO3) to trimethylamine N-oxide (TMAO), and elevated plasma TMAO levels have been consistently identified as an independent risk factor associated with the progression and poor prognosis of various systemic diseases, including atherosclerosis, thrombosis, and chronic kidney disease [8,9,10].

At the cellular level, TMAO induces endothelial dysfunction by activating the NLRP3 inflammasome via the ROS-TXNIP pathway, leading to caspase-1 activation and increased secretion of IL-1β and IL-18. It concurrently inhibits eNOS phosphorylation, thereby reducing NO bioavailability [11]. In macrophages, TMAO upregulates scavenger receptors (CD36, SR-A1) through the MAPK/JNK pathway, enhancing oxidized low-density lipoprotein (ox-LDL) uptake and promoting foam cell formation [12]. In terms of thrombosis, TMAO directly interacts with platelets, enhancing intracellular Ca^2+^ release, which primes platelets for hyperreactivity and promotes thrombus formation [13]. This mechanism may contribute to the acceleration of various cardiovascular and cerebrovascular diseases, such as atherosclerosis and ischemic stroke [14,15,16,17,18,19,20]. In *ApoE*^−/−^ mice, dietary supplementation with choline or TMAO accelerates the development of aortic plaques, increases macrophage infiltration, and elevates levels of pro-inflammatory cytokines (IL-6, TNF-α). Notably, these effects are abrogated by antibiotic treatment or inhibition of the CutC enzyme, confirming the gut microbiota-dependent nature of this pathway [21]. In FeCl_3_-induced thrombosis models, mice with elevated plasma TMAO exhibit a significantly shortened time to arterial occlusion, demonstrating a direct pro-thrombotic phenotype [13,22]. Data from large-scale human prospective cohort studies, such as EPIC-Norfolk and CNSR-III, confirm a significant and independent association between elevated TMAO levels and the risk of cardiovascular and cerebrovascular diseases, even after adjusting for traditional risk factors [8,9,23]. Consequently, TMAO is considered an important biomarker for diagnosing and predicting atherosclerotic cardiovascular and cerebrovascular diseases [24], and reducing circulating TMAO levels has emerged as a key strategy for treating these conditions [25].

**Figure 1:**
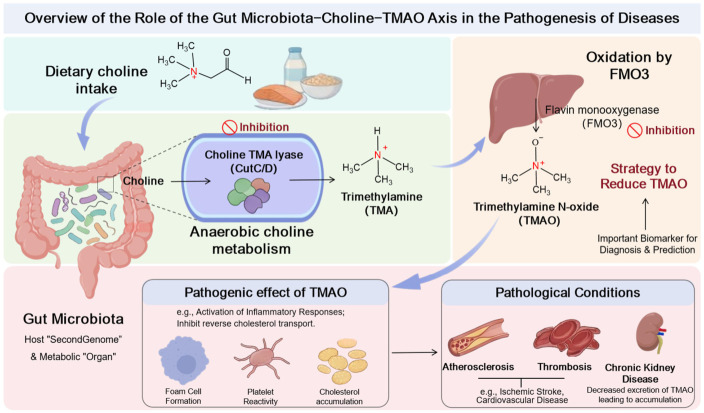
**Overview of the role of the gut microbiota–choline–TMAO axis in the pathogenesis of diseases.** Dietary choline is metabolized by the gut microbiota via choline TMA lyase (CutC/D) to trimethylamine (TMA), which is then oxidized by hepatic flavin monooxygenase 3 (FMO3) to trimethylamine N-oxide (TMAO). The circulating TMAO may promote inflammation, cholesterol accumulation, foam cell formation, and platelet hyperreactivity, thereby contributing to various metabolic diseases such as atherosclerosis, thrombotic events, and chronic kidney disease. It is an important biomarker for diagnosis and prediction. Therefore, inhibiting the key enzymes of this metabolic axis, such as the microbial CutC/D or the host hepatic FMO3, represents a major therapeutic strategy currently under investigation to lower circulating TMAO levels.

The choline–TMA–TMAO metabolic pathway involves two key enzymes: the microbial choline TMA-lyase (CutC), responsible for the first step of choline-to-TMA conversion, and the host’s liver-endogenous enzyme FMO3, responsible for the second step of TMA-to-TMAO conversion. Interventions to inhibit these key enzymes can directly or indirectly reduce circulating TMAO concentrations. Reports indicate that genetic defects or inhibition of liver FMO3 can lead to liver injury, such as hepatitis [26]. Furthermore, when TMA cannot be metabolized, it accumulates in the body, leading to a fishy odor in the patient’s breath, sweat, and urine, a condition known as trimethylaminuria (fish odor syndrome), which impacts daily life [27]. In conclusion, targeting CutC, the enzyme responsible for the initial conversion, offers a promising therapeutic strategy. It can reduce TMA production at the source, thus lowering the TMAO burden on the body and providing a novel treatment approach for cardiovascular and cerebrovascular diseases (Figure 1).

## 2. Discovery and Biogenetic Characteristics of Choline TMA-Lyase CutC

### 2.1. Discovery of the Cut Gene Cluster

The bacterial conversion of choline to TMA was first reported in 1910, primarily involving anaerobic choline metabolism by gut microorganisms symbiotic with humans and other vertebrates. The volatile TMA produced can also be used as a carbon source by bacteria, for instance, being converted to methane, a potent greenhouse gas, by archaea in soil and the marine environment [28]. In 2012, Balskus and colleagues successfully revealed the biogenetic characteristics of microbial anaerobic choline metabolism to generate TMA [29]. They discovered a gene cluster responsible for choline utilization in *Desulfovibrio desulfuricans* and characterized the function of the metabolic enzymes involved in TMA production [29,30,31,32,33]. Microbial anaerobic choline metabolism is a process that cleaves the choline C-N bond to produce TMA and acetaldehyde. This is chemically similar to ethanolamine degradation in bacteria, which cleaves a C-N bond to produce ammonia and acetaldehyde [34]. The enzymes required for ethanolamine utilization are encoded by the *eut* gene cluster [35]. Hypothesizing that the microbial conversion of choline is analogous to the downstream conversion of ethanolamine’s product, acetaldehyde, Balskus and colleagues used position-specific iterative BLAST (PSI-BLAST) [29] to search for genes homologous to the *eutG*, *eutE*, and *eutM* microcompartment protein-encoding genes of *Salmonella enterica* in *Desulfovibrio desulfuricans*, which was known to metabolize choline to TMA. They identified a clustered group of genes with a structural organization similar to the *eut* gene cluster, which they named the choline utilization (*cut*) gene cluster [29,36].

The cut gene cluster contains 19 open reading frames (ORFs) [36], which are protein-coding gene regions. Unlike the *eut* gene cluster, homologs of the *eutB/C* genes that encode ethanolamine ammonia-lyase are not found among these 19 ORFs or in other *Desulfovibrio desulfuricans* genomes. Instead, a unique set of genes, *cutC/D*, annotated as glycyl radical enzyme CutC and glycyl radical activating protein CutD, is responsible for cleaving the choline C-N bond to produce TMA and acetaldehyde. In addition to the TMA-producing enzymes CutC/D, the cut gene cluster encodes other functional proteins for choline metabolism. Eight genes encode proteins for the bacterial microcompartment (BMC) shell, including *cutA/E/G/K/L/N/Q/R*. Choline traverses the BMC shell, and inside the compartment, it is first cleaved by CutC/D to generate TMA and acetaldehyde. The BMC shell sequesters the acetaldehyde [37,38,39], a substance harmful to bacteria, and processes it to provide energy, involving two predicted CoA-acylating aldehyde oxidoreductases (CutB/F) and one phosphate acetyltransferase (CutH). The cut gene cluster encodes functional proteins for all steps of intramicrobial choline metabolism. These proteins aggregate to form the intramicrobial site of choline utilization, a microcompartment (BMC) that encapsules the catalytic cleavage of choline. This assists in identifying the intracellular location of the enzymes and underscores the crucial role of the cut gene cluster in the microbial anaerobic metabolism of choline to TMA. Further heterologous expression and gene knockout experiments confirmed that the CutC/D enzymes are essential for anaerobic microbial choline metabolism; strains with *cutC/D* knocked out were unable to grow on choline-containing media [29]. Research also demonstrated that the catalytic core, CutC, strictly relies on the synergistic action of the activating protein CutD, with both forming the complete functional unit for anaerobic choline metabolism.

### 2.2. Phylogenetic Distribution and Ecophysiological Characteristics of CutC in the Gut Microbiota

The human gut is a major site where the cut gene cluster is distributed and functional. Early studies combining culturomics and genomics first systematically revealed the diversity of TMA-producing bacteria in human feces [40]. They demonstrated that the cut gene cluster, particularly the *cutC/D* genes, is widely distributed in the human gut microbiome, spanning the phyla *Firmicutes*, *Proteobacteria*, and Actinobacteria [36,41]. Examples include species such as *Clostridium sporogenes*, *Klebsiella pneumoniae*, *Proteus mirabilis*, and *Escherichia coli*. Metagenomic data mining within the *Firmicutes* phylum identified multiple members of the class *Clostridia* as key TMA-producing gut microbes, particularly many uncultured or difficult-to-culture bacteria in the order *Clostridiales*, where members of the families *Lachnospiraceae* and *Ruminococcaceae* have been confirmed as potent TMA producers [42]. On the other hand, the *cutC* gene is detected in over 95% of human gut samples, but its relative abundance is generally low, typically accounting for less than 1% of the community [42,43]. However, this low abundance does not diminish its functional significance. Researchers observed in a germ-free mouse model that colonization with *Clostridium sporogenes*, a CutC-positive bacterium [44], led to a significant increase in serum TMAO levels and platelet reactivity, despite accounting for only about 0.2% of the gut community. Microbial transplant experiments proved that colonization with a strain possessing a loss-of-function mutation in the *cutC* gene completely eliminated the effects of increased host TMAO levels and enhanced thrombosis caused by CutC-positive bacteria [24]. This further confirms that CutC is the functional key point for choline metabolism to generate TMA. Metagenomic association study (MWAS) further found that urinary TMAO levels are significantly positively correlated not only with the overall composition of the gut microbiota but also with the abundance of the cutC gene carried by *Enterobacteriaceae* [45], suggesting that certain specific cutC-containing microbial species may play a dominant role in TMAO production. From an ecological perspective, TMA-producing bacteria are not uniformly distributed throughout the gut. Buckley et al. [46] used an in vitro simulated system to confirm that the distal colon is the primary site for choline-to-TMA conversion, related to the lower partial pressure of oxygen, longer content residence time, and specific microbial community structure in that region. These existing research findings suggest that the CutC enzyme is a key node in the gut microbiota–host interaction, not dependent on abundance. Therefore, intervention strategies targeting the gut microbial CutC enzyme should focus on the functional activity of the strain rather than simple abundance regulation to more precisely prevent multiple disease risks, such as cardiovascular and cerebrovascular diseases.

## 3. Structural Characteristics and Catalytic Mechanism of CutC

**Figure 2:**
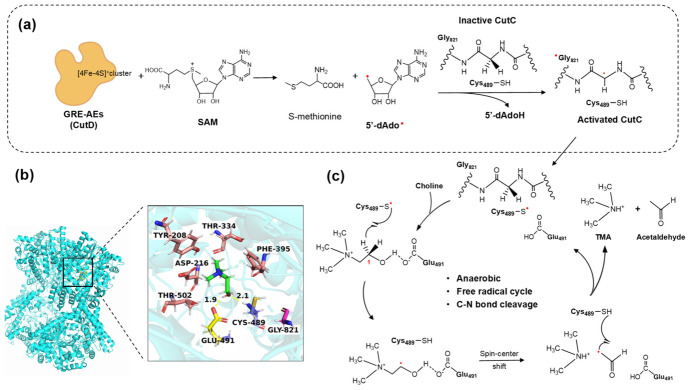
**The molecular mechanism of anaerobic choline metabolism catalyzed by choline trimethylamine lyase**. (**a**) The reaction mechanism by which CutD activates the CutC enzyme; (**b**) the crystal structure and active site of the wild-type CutC enzyme in complex with the substrate choline (PDB:5FAU); Key residues and the substrate are shown as sticks and colored as follows: green, choline; yellow, Glu491; purple, Cys489; pink, Gly821, red, other amino acid residues in the active pocket. (**c**) Under anaerobic conditions, through a series of free radical reactions, the CutC enzyme catalyzes the anaerobic cleavage of the C-N bond in choline to generate TMA and acetaldehyde. Red dots represent free radicals; Mark the position of the substrate choline C1 with the red number 1.

The identity of choline TMA-lyase CutC as a member of the glycyl radical enzyme (GRE) family has been fully confirmed. GREs are important biocatalysts for strictly anaerobic and facultatively anaerobic bacteria, playing crucial roles in microbial anaerobic metabolism [47]. Members of the GRE family are isozymes, characterized by an active site containing a glycyl radical that utilizes a protein-based radical intermediate to catalyze diverse reactions, including C-C bond and C-O bond cleavage and C-C bond formation. CutC’s catalysis of choline C-N bond cleavage is a novel function of GREs. The common structural features of GREs that CutC shares include a 10-α/β-barrel structure and conserved glycine (Gly) and cysteine (Cys) residues. The GRE active site, composed of a Gly ring and a Cys ring, is located between two five-stranded half-barrels arranged anti-parallel, with an external α-helix. In the active state, the Gly and Cys rings are juxtaposed, facilitating radical transfer. To allow the GRE activating enzyme (GRE-AE) to perform initial H-atom abstraction, the barrel conformation must change to an “open” state [48], exposing the hidden internal Gly residue for GRE activation, forming a glycyl-centered radical [49]. GRE-AEs are a group of S-adenosylmethionine (SAM) radical superfamily enzymes containing a [4Fe-4S]^+^ cluster, typically encoded adjacent to GREs in microbial genomes [50]. GRE-AEs feature a conserved CX3CX2C motif that coordinates the [4Fe-4S]^+^ cluster. Using the reduced [4Fe-4S]^+^ to provide an electron to the SAM substrate, SAM is reductively cleaved to form S-methionine and a 5’-deoxyadenosyl radical (5’-dAdo**·**). The 5’-dAdo**·** acts as a radical donor to extract the H-atom from the conserved and catalytically essential Gly of the GRE, forming the glycyl radical and initiating subsequent radical reactions (Figure 2a) [47].

Marina et al. [51] proposed that the glycyl radical of CutC abstracts an H-atom from the adjacent cysteine, Cys489, generating a thiyl radical intermediate that initiates the reaction with choline. Within the polar active site of CutC, choline is oriented such that its C1-hydroxyl forms a hydrogen bond with Glu491, stabilizing enzyme–substrate binding. Substrate specificity is maintained by a series of CH-O hydrogen bonds between the polarized methyl groups of the trimethylammonium moiety and the side-chain oxygen atoms of Asp216, Try208,Thr334, Thr502, Try506 and Phe395, as well as bound water molecules [32] (Figure 2b). The thiyl radical first abstracts an H-atom from the C1 of choline, followed by deprotonation of the C1-OH by Glu491, causing C-N bond cleavage via “spin-center shift” to eliminate TMA. The resulting product-based radical can re-abstract a hydrogen atom from Cys489, regenerating the ethyl radical and generating acetaldehyde. Glu491-mediated proton transfer resets the radical in the CutC active site for the next round of catalysis (Figure 2c) [30,52]. A unique aspect of this catalytic process is its strict anaerobic dependence. Experimental studies have shown that CutC catalytic activity significantly decreases when the oxygen concentration exceeds 2%, closely related to the oxygen-sensitive radical intermediate at the active center [33,53]. More importantly, the participation of the CutD enzyme is essential for the catalytic reaction. Research found that without the CutD enzyme, CutC exhibits no significant catalytic activity even at high substrate concentrations. This was verified by gene knockout experiments where an engineered strain with cutD knocked out completely lost choline metabolism, while complementing the *cutD* gene restored its function [29,30,54]. The current revelation of the reaction mechanism and key catalytic active site of CutC for choline metabolism provides a core design theory and solid structural foundation for the development and discovery of CutC inhibitors.

## 4. From Mechanism to Therapy: Implications and Challenges of the TMAO Causality Hypothesis

Based on the deep understanding of the structure and catalytic mechanism of the CutC enzyme described above, a molecular foundation has been laid for the rational design of its inhibitors. However, the fundamental prerequisite for establishing the gut microbial CutC enzyme as a therapeutic target lies in confirming the core role of its product, TMAO, in disease. The therapeutic strategy of targeting CutC is based on a central hypothesis: reducing TMAO levels will yield clinical benefits. Therefore, it is crucial to critically examine the evidence linking TMAO to disease. A large body of human observational cohort studies has consistently shown that elevated circulating TMAO is a strong, independent risk factor for major adverse cardiovascular events, chronic kidney disease progression, and other conditions [8,9,23,24]. Mechanistic studies in animal models and cell cultures have provided plausible biological pathways, demonstrating that TMAO can promote inflammation, foam cell formation, and endothelial dysfunction [11,12,13,21,22].

However, the direct causal role of TMAO in the pathogenesis of human diseases remains an area of active scientific debate [55]. Key considerations include: (1) the limitations of extrapolating conclusions from animal studies that often use supraphysiological doses of TMAO to human physiology; (2) the possibility that TMAO may be a marker of impaired renal function (its primary clearance route) or overall metabolic disturbance rather than a primary driver; and (3) the existence of studies suggesting neutral or even context-dependent protective effects of TMAO or its precursors [56,57]. Furthermore, the relationship between dietary choline intake and plasma TMAO levels in humans is not linear and is significantly modulated by individual factors such as gut microbiota composition, liver FMO3 activity, and renal efficiency [55].

These nuances reveal a fundamental therapeutic implication: if TMAO is primarily a biomarker of underlying pathology (such as renal impairment or specific dysbiosis), then inhibiting its production via the CutC enzyme may have limited clinical efficacy. Conversely, if TMAO is an active pathogenic agent, then targeted inhibition holds great promise. This distinction highlights the paramount importance of future research employing Mendelian randomization, long-term intervention trials, and detailed human mechanistic studies to definitively establish causality. It is precisely within this context of coexisting opportunities and challenges that the following section will systematically review the current intervention strategies targeting CutC. Their development essentially constitutes a strategic exploration of this core hypothesis.

## 5. Intervention Strategies and Inhibitor Development Targeting CutC

### 5.1. Dietary Intervention

**Figure 3:**
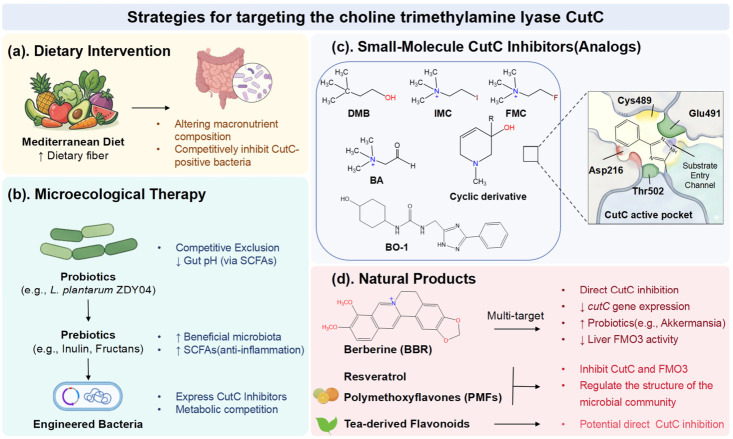
**Strategies for targeting the choline trimethylamine lyase CutC.** Four principal approaches to reduce TMAO levels by targeting choline trimethylamine-lyase (CutC) are depicted: (**a**) dietary modulation (e.g., Mediterranean diet); (**b**) microbiota-directed therapy (e.g., probiotics, prebiotics, engineered bacteria); (**c**) direct small-molecule inhibition (e.g., DMB, FMC/IMC, BA); (**d**) multi-target natural products (e.g., berberine, resveratrol, flavonoids).

The gut microbial choline TMA-lyase CutC, acting as the key rate-limiting enzyme in choline metabolism, is a critical target for interfering with the “diet–gut–host” axis. As a foundational modulatory approach, dietary intervention primarily reshapes the gut microbial metabolic environment by altering overall dietary patterns (e.g., increasing dietary fiber, specific phytochemicals), thereby indirectly influencing CutC-mediated TMA production (Figure 3a). This includes introducing potential CutC inhibitory components or modulating the metabolic fate of choline by altering microbial community structure. While simply limiting precursor choline intake is a direct mechanism, its potential impacts on host nutritional status and long-term health benefits remain unclear and require cautious consideration. Notably, inappropriate dietary choline restriction may lead to choline deficiency, which has been associated with an increased risk of metabolic dysfunction-associated steatotic liver disease (MASLD) [58] and cognitive impairment [59]. Dietary patterns can influence TMAO generation through complex mechanisms. For instance, diets rich in red meat and processed foods may selectively enrich specific CutC-positive bacterial taxa. Conversely, diets abundant in dietary fiber, such as the Mediterranean diet, may indirectly reduce TMAO exposure by promoting the growth of short-chain fatty acid-producing bacteria, which can competitively inhibit CutC-positive bacteria, and by enhancing intestinal barrier function [60,61]. Simultaneously, SCFAs like butyrate can enhance gut barrier function, reducing TMA transport from the gut lumen to the portal vein [62]. Beyond overall dietary patterns, restriction or supplementation of specific nutrients also regulates the choline–TMA–TMAO axis. A methionine-restricted diet in a C57BL/6J mouse model alleviated choline-induced TMAO elevation [63]. However, it is important to note that directly restricting dietary choline intake as a strategy to lower TMAO lacks support from epidemiological evidence regarding its long-term safety and efficacy, and may carry risks associated with choline deficiency. Therefore, it should not be considered a universal therapeutic recommendation. The mechanism involves gut microbiota remodeling by reducing the relative abundance of *cutC*-high-expressing bacteria while increasing the proliferation of beneficial bacteria like *Lactobacillus*. This modulatory effect might be related to S-adenosylmethionine (SAM) produced by methionine metabolism, which acts as a major methyl donor in bacterial epigenetics and could affect the methylation status and expression level of the *cutC* gene [50]. However, the efficacy of dietary intervention is subject to individual microbial composition, genetic background, and poor long-term adherence.

### 5.2. Gut Microecological Therapy

Gut microecological therapy directly modulates microbiota composition or function to interfere with CutC activity, primarily utilizing antibiotics, probiotics, and prebiotics to reshape the gut ecosystem and reduce the abundance and activity of CutC-positive bacteria (Figure 3b). Broad-spectrum antibiotics like vancomycin and neomycin can rapidly reduce TMA-producing bacteria and significantly lower plasma TMAO levels in the short term. However, long-term use leads to severe issues. The non-selective action of antibiotics disrupts gut microecological balance, eliminating both CutC-positive and beneficial bacteria, leading to reduced diversity and functional disorder. This imbalance can cause antibiotic-associated diarrhea, *Clostridioides difficile* infection, and other complications, with long-term use likely leading to resistance, making this strategy undesirable. In contrast, selective inhibition of CutC-positive bacteria or modulation of their metabolism is a safer, more effective approach. Probiotics show distinct advantages. *Lactobacillus plantarum* ZDY04 in a C57BL/6J mouse model exhibited a strain-specific effect in reducing TMAO [64] through competitive inhibition of CutC-positive bacterial growth and reduction in choline-to-TMA metabolism. Similarly, a combined intervention of *Bifidobacterium breve* and *Bifidobacterium longum* attenuated choline-induced plasma TMAO elevation by modulating the gut microbiota [65]. These probiotics may increase short-chain fatty acids like acetate and propionate, lowering gut pH and inhibiting CutC-positive bacterial proliferation, while promoting a thickened mucus layer and tight junction protein expression to enhance gut barrier integrity. Prebiotics, as non-digestible food components, selectively stimulate the growth and activity of beneficial bacteria, indirectly inhibiting CutC-positive bacterial growth. For example, a randomized cross-over trial of inulin-type fructans in peritoneal dialysis patients showed a significant reduction in plasma TMAO after 12 weeks [66]. The mechanism might be that short-chain fatty acids (SCFAs) from inulin fermentation, especially butyrate, lower gut pH to inhibit CutC-positive bacteria; additionally, inulin promotes beneficial bacteria like *Bifidobacterium* and *Lactobacillus*, which can inhibit CutC-positive bacteria through antimicrobial substances or competitive exclusion. Beyond traditional probiotics and prebiotics, microbial transplant (e.g., fecal microbiota transplant) and engineered bacterial therapies also show potential to regulate the gut microbial CutC enzyme–TMAO axis. Research indicates that transplanting *cutC*-containing microbiota is sufficient to enhance platelet reactivity and thrombosis potential [44], suggesting that selective transplantation of *cutC*-negative or low-expressing microbiota might reverse TMAO-related pathology. Engineered bacterial therapy can design bacteria to express CutC inhibitors or compete for choline via other metabolic pathways for more precise intervention. However, these new therapies are in early research stages, and their safety, efficacy, and long-term stability require further verification. In clinical translation, microecological therapy faces challenges like large individual differences, complex mechanisms, and standardization difficulties. A future need exists for personalized microecological intervention plans based on multi-omics, combining metagenomic sequencing, metabolomic analysis, and host genotype to predict an individual’s response and achieve precise regulation.

### 5.3. Small-Molecule Inhibitors of CutC

Small-molecule inhibitors directly target the CutC active center or allosteric sites to block its catalysis and are currently the most promising strategy for precise intervention to lower TMAO. Crystal structure analysis reveals that the CutC active pocket contains conserved residues like Cys489, Glu491, Asp216, Try208, Thr334, Thr502, Try506 and Phe395 [30,32,52], which bind the substrate choline via hydrogen bonds and hydrophobic interactions. This research provides a key structural basis for finding interacting molecules. System evolutionary analysis shows high CutC conservation in TMA-producing bacteria and wide existence in the human gut, supporting its feasibility as a broad-spectrum target. Compared to diet and microecological therapy, small-molecule inhibitors are direct, efficient, dose-controllable, and easy to standardize. Based on mechanism and structure, CutC inhibitors are categorized into substrate analogs and natural product-based inhibitors, each with unique chemical features and action modes.

#### 5.3.1. Substrate Choline Analogs

Substrate analogs were the first class of CutC inhibitors developed, designed based on choline’s structural features to competitively bind the active center without being catalyzed (Figure 3c and Table 1). Wang et al. [67] reported the first CutC inhibitor, 3,3-dimethyl-1-butanol (DMB), which replaces the choline nitrogen with a carbon atom, and proved that oral DMB lowers mouse plasma TMAO levels. In an atherosclerosis mouse model, DMB treatment reduced high-choline-diet-induced formation of endogenous macrophages and foam cells and alleviated atherosclerotic lesions in *ApoE***^−/−^** mice. However, in bacterial culture, DMB showed weak CutC inhibition, possibly due to substance interactions in the complex in vivo environment, and might suffer from low oral bioavailability and metabolic stability, limiting clinical application. Another class, halo-choline analogs like fluoromethylcholine (FMC) and iodomethylcholine (IMC) [22,68], inhibits enzyme activity via irreversible binding. Co-incubation with human gut commensals showed IMC and FMC are non-lethal inhibitors, only inhibiting CutC without affecting strain growth, an ideal characteristic. In mouse models, FMC and IMC lowered plasma TMAO long-term, but their efficacy and safety as atherosclerosis treatments need further clinical evaluation.

Leveraging a deep understanding of the CutC mechanism, Balskus and colleagues developed and screened highly active CutC inhibitors, betaine aldehyde (BA) and choline cyclic derivatives [51,52]. BA effectively inhibited choline-to-TMA metabolism in multiple whole-cell experiments of choline-degrading human gut bacteria [52]. Based on mechanism and structural information, researchers continuously optimized compound structures, including nitrogen ring size, hydroxyl position, and replacing the hydroxyl with a carbonyl to enhance hydrogen bonding with the active center, improving inhibitory activity and metabolic stability, ultimately finding a cyclic choline analog with effective CutC inhibition [51]. However, these structure-guided inhibitors remain in the in vitro stage, lacking pharmacological experiments, reflecting synthesis challenges and the need to address stability, specificity, and safety in inhibitor development.

Gabr et al., by screening peptidomimetic libraries and assessing gut metabolic stability, broke the limitations of traditional analogs, finding compound 5 (IC_50_ ≈ 5.9 μM) as a non-competitive CutC inhibitor [69]. It effectively inhibited TMA production in *Escherichia coli*, *Clostridium* spp., and other TMA-producing strains, and human fecal suspensions, without significantly affecting bacterial growth. They also discovered a histidine scaffold-based inhibitor with competitive inhibition, with compound 5 (IC_50_ ≈ 1.9 μM) not only blocking CutC catalysis but also downregulating liver FMO3 mRNA expression, maintaining high inhibition in both cells and complex biological environments [70]. Another study focused on a glycomimetic backbone, designing a benzoxazole derivative BO-I (IC_50_ ≈ 2.4 μM) as a non-competitive inhibitor [71]. Molecular dynamics modeling showed it binds to the CutC substrate entry channel, hindering choline recognition and binding, outperforming traditional cyclic choline analogs in multiple strains and human fecal samples, providing a clear direction for optimization.

#### 5.3.2. Natural Products

Natural products are another vital source of CutC inhibitors due to low toxicity and multi-target regulation (Figure 3d and Table 2). Berberine (BBR), an isoquinoline alkaloid, is one of the most studied. Multiple studies confirm BBR reduces TMAO levels and improves associated disease phenotypes through multiple mechanisms. Ma et al. found BBR interferes with the choline–TMA–TMAO metabolic pathway [54], exerting a vitamin-like regulatory function to directly target and inhibit the activity of CutC enzyme in the gut microbiota, reducing TMA/TMAO generation, thereby inhibiting thrombosis. In vitro enzyme inhibition assays and molecular dynamics simulations have similarly confirmed that BBR can occupy the active pocket of the CutC enzyme, forming a stable complex that inhibits the binding of the enzyme to its substrate, choline, thereby suppressing TMA production. Nevertheless, it cannot be overlooked that berberine possesses potent antimicrobial activity, suggesting that its dominant in vivo effect may primarily manifest as a remodeling of the overall gut microbiota structure. BBR regulates gut microbiota structure, increasing beneficial *Akkermansia* abundance, downregulating microbial *cutC* gene expression, and reducing TMA production, while also regulating host liver FMO3 activity to reduce TMA-to-TMAO conversion, achieving multi-target regulation [54,72]. Stilbene derivatives are another class of cardio-protective natural products with emerging CutC inhibitory potential: molecular docking shows resveratroloside has the strongest affinity for CutC [73], and its aglycone resveratrol can also reduce TMAO via dual mechanisms of inhibiting *cutC* gene expression and regulating the FMO3 pathway, while also modulating gut microbiota (e.g., increasing *Lactobacillus* and *Bifidobacterium*, decreasing *Bacteroidetes*) and promoting bile acid metabolism, indirectly lowering TMAO. It has demonstrated significant myocardial protection in an atherosclerosis model [73,74]. Polymethoxyflavones (PMFs) from citrus are a hot research topic. 3,6,7,8,2’,5’-hexamethoxyflavone [75], isolated from Valencia orange peel, reduces TMA production by inhibiting the *cutC/D* pathway while downregulating hepatocyte FMO3 mRNA, reducing TMA-to-TMAO conversion. Structure–activity relationship analysis indicates that the A-ring methoxy group enhances inhibitory activity via hydrogen bonding, and further methoxy substitution can strengthen binding through increased hydrophobicity. Tea-derived flavonoids also possess CutC inhibitory activity. Researchers found kaempferol 3-O-rutinoside from Lu’an GuaPian tea had the highest Vina score of all tested flavonoids [76], potentially preventing coronary heart disease by blocking CutC activity, inhibiting inflammatory signaling, and regulating gut microecology. These natural products not only directly inhibit CutC catalysis but also modulate the gut–liver axis multi-dimensionally, offering intervention strategies more akin to the physiological environment for TMAO-related diseases.

### 5.4. Summary of Clinical and Preclinical Evidence for TMAO Reduction

To provide a comprehensive overview of the current interventional landscape targeting the TMAO pathway, we have summarized key clinical and preclinical studies focusing on dietary modifications, microbiome modulations, and natural products (Table 3). Our analysis indicates that dietary strategies, particularly the Mediterranean diet with restricted red meat intake, consistently demonstrate significant reductions in both fasting and postprandial TMAO levels. Conversely, while some microecological interventions, such as synbiotics, showed no significant impact on TMAO in certain high-risk cohorts, others like probiotics exhibited a favorable downward trend. Notably, natural products such as Berberine have shown promise, with clinical data indicating substantial decreases in TMAO levels (up to 35%) alongside improvements in vascular function. This synthesis highlights the translational potential of these strategies, although the variability in study outcomes underscores the need for standardized methodologies and larger, long-term clinical trials to validate these findings.

## 6. Conclusions and Outlook

As a core node connecting the “diet–microbiota–host” metabolic axis, the gut microbial choline TMA-lyase CutC has emerged as a promising and potentially pivotal therapeutic target for diseases associated with elevated Trimethylamine N-oxide (TMAO) levels. This review has systematically summarized the discovery of CutC, its unique glycyl radical catalytic mechanism, and its critical role in the pathogenesis of host diseases. Substantial evidence identifies CutC as the rate-limiting enzyme for microbial choline-to-TMA conversion, directly determining the systemic TMAO concentration. Given the pathological involvement of TMAO in atherosclerosis, thrombosis, chronic kidney disease, and neurodegenerative disorders, targeting CutC serves not only as an effective means to inhibit TMAO production but also as a novel upstream strategy for preventing and treating systemic diseases.

CutC presents several distinct advantages as a therapeutic target. First, it provides high precision. Unlike the direct inhibition of host hepatic FMO3, which may lead to TMA accumulation (e.g., trimethylaminuria) or hepatic injury [26,27], targeting CutC specifically blocks intestinal TMA production without interfering with the host’s endogenous choline metabolism. Second, it exhibits high conservation. The *cutC* gene and its cluster are highly conserved across various TMA-producing gut bacteria [36], suggesting that the development of broad-spectrum CutC inhibitors holds promise, although their efficacy may vary depending on individual gut microbiota composition and ecological context. Furthermore, the mechanism is well-defined. The complex structure formed by CutC and its activating protein CutD, along with the detailed radical mechanism of C-N bond cleavage (e.g., glycyl radical transfer and conformational changes in the substrate-binding pocket) [30,51,52], provides a robust theoretical foundation for structure-based rational drug design.

Progress has been made in the development of CutC inhibitors. Design strategies for substrate analogs targeting the CutC active pocket have achieved certain results, evolving from early 3,3-dimethyl-1-butanol (DMB) to cyclic choline analogs, peptidomimetics, and histidine-scaffold-based inhibitors. While inhibitory potency has been improved in vitro for some lead compounds, their development concurrently faces significant scientific and translational hurdles. Simultaneously, natural products demonstrate immense clinical potential due to their multi-target regulation and low toxicity. Alkaloids like berberine can occupy the CutC active center and regulate bile acid metabolism and gut microbiota structure simultaneously [72]. Citrus flavonoids (e.g., polymethoxyflavones) bind stably to CutC through hydrogen-bond networks and hydrophobic interactions, exerting multi-level pharmacological effects including anti-inflammation and endothelial protection. These studies confirm the feasibility of developing CutC inhibitors with high oral bioavailability and colonic targeting through chemical modification and bioactive screening of small molecules from traditional Chinese medicine (TCM). However, while many TCM compounds reduce TMAO by broadly remodeling the microbiota, such non-specific regulation may lead to dysbiosis and metabolic disturbances.

Currently, the development of inhibitors targeting gut microbial CutC faces significant scientific challenges and translational hurdles. The primary obstacle lies in selectivity: most known compounds lack sufficient specificity for CutC, carrying the risk of cross-reactivity with host homologs (e.g., enzymes involved in phosphatidylcholine metabolism). This could not only disrupt the host’s normal one-carbon metabolism and methyl donor balance but also lead to unintended perturbations in the overall structure and function of the gut microbiota. Secondly, delivery and stability present another critical bottleneck: oral drugs must maintain stability in the complex gastrointestinal environment and be effectively delivered to the anaerobic niche of the colon to exert inhibitory activity, imposing stringent demands on the compound’s chemical properties and formulation design. Furthermore, heterogeneity at the microbiome level contributes to efficacy uncertainty: functional and expressional variations in the CutC enzyme among different individuals and even bacterial strains may result in a lack of universal inhibitory effect. Additionally, the long-term safety and drug resistance remain a largely unexplored area: while no significant toxicity has been observed in short-term interventions, the potential consequences of long-term blockade of the microbial choline metabolism pathway—such as functional compensation within the microbiome, shifts in nutritional competition dynamics, or impacts on host choline utilization—require systematic evaluation through long-term toxicity studies in large animal models and rigorous clinical trials.

To address these challenges, future research and development should adopt a multi-dimensional strategy. At the drug discovery level, deep integration of structural biology and computational chemistry is essential. Rational design of next-generation inhibitors with high potency and selectivity should be based on the intricate three-dimensional structure of CutC and its interaction patterns with substrates or inhibitors. At the technology-enablement level, Artificial Intelligence-Aided Drug Design (AIDD) can significantly enhance the efficiency of virtual screening and optimization of lead compounds. The application of nanodelivery systems, prodrug strategies, or colon-targeted formulations holds promise for precisely overcoming delivery challenges like low oral bioavailability and insufficient colonic drug concentration. At the clinical translation level, a comprehensive in vitro and in vivo evaluation system must be established, encompassing enzyme inhibition, cellular toxicity, microbiome impact, and efficacy and safety validation in animal models. More importantly, therapeutic strategies must evolve towards precision and personalization: by integrating multi-omics data, such as metagenomics (assessing *cutC* gene abundance and strain distribution) and metabolomics (monitoring TMAO and precursor dynamics), precise patient stratification can be achieved. This will enable the identification of specific subpopulations most likely to benefit from CutC inhibitor therapy, realizing the goal of “precision microbiome modulation.”

In conclusion, targeting the gut microbial CutC enzyme opens an attractive new avenue for intervening in TMAO-related metabolic diseases. Its core vision is to achieve precise regulation of specific microbial metabolic functions, rather than broadly altering the microbial community structure. With deepening understanding of CutC enzymology, microbial ecology, and host–microbe interactions, coupled with the convergence of interdisciplinary technologies, it is possible to develop effective and selective microbiome-modulating therapies targeting CutC. This effort paves the way for their translation from concept toward clinical application, offering novel solutions for the prevention and treatment of cardiovascular, renal, and metabolic diseases.

## Figures and Tables

**Table 1:** Substrate analog-based CutC inhibitors: structure-guided design and mechanism of action.

Compound/ Class	Mechanism of Action	In Vitro Potency/ Key Features	In Vivo Evidence (Model, Effect)	Limitations	References
DMB (3,3-Dimethyl-1-butanol)	Non-lethal inhibition of bacterial CutC.	Moderate potency, significant differences exist among different strains (IC_50_ > 1 mM)	Animal models: Significantly reduced plasma TMAO; attenuated atherosclerotic plaque formation without altering cholesterol levels.	Moderate potency, requires long-term intervention; clinical translation potential is still under evaluation.	[67]
FMC/IMC (Fluoro/Iodomethylcholine)	Mechanism-based/irreversible inhibitor.	Potent (superior to DMB) (IC_50_ ≈ 1.4 nM)	Animal models: Potently lowered TMAO, inhibited platelet hyperreactivity, and reduced thrombosis risk.	Difficult synthesis; potential off-target toxicity; currently in preclinical development.	[22,68]
BA (Betaine Aldehyde)	Mechanism-based design.	Effective in whole-cell experiments of multiple human gut bacteria (IC_50_ ≈ 26 μM)	No clear in vivo pharmacodynamic data reported.	Metabolically unstable (easily oxidized to betaine); poor drug-like properties.	[52]
Cyclic Choline Analogs	Based on CutC mechanism and structural information optimization.	Optimized analogs showed effective CutC inhibitory activity (IC_50_ ≈ 2.9 μM)	No clear in vivo pharmacodynamic data reported.	Synthesis is challenging, stability, specificity, and safety issues need to be addressed.	[51]
Peptidomimetic Compound 5	Non-covalent binding of the active site of CutC, blocking choline binding.	Effective in multi-strain and fecal suspension solutions (IC_50_ ≈ 5.9 μM)	No clear in vivo pharmacodynamic data reported.	Requires further structural optimization.	[69]
Histidine scaffold-based inhibitor	Competitive inhibitors occupy the substrate pocket of CutC.	In a complex microbial community environment, it can significantly reduce TMA (IC_50_ ≈ 1.9 μM)	No clear in vivo pharmacodynamic data reported.	Limited exploration of chemical space.	[70]
BO-I (Benzoxazole)	Non-competitive inhibitors bind to the allosteric sites of the non-active center of the enzyme.	Effective in multi-strain and fecal suspension solutions (IC_50_ ≈ 2.4 μM)	No clear in vivo pharmacodynamic data reported.	The physiological relevance of the non-competitive mechanism remains unclear.	[71]

**Table 2:** Natural product-derived CutC inhibitors: evidence levels, mechanisms of action, and key findings.

Compound/ Class	Primary Mechanism of Action Targeting CutC/TMAO Pathway	Level of Evidence for CutC/TMAO Inhibition	Key Experimental Findings (In Vitro/In Vivo/Clinical)	Limitations	References
Berberine	1. Direct Inhibition: Its gut microbial metabolite dihydroberberine (dhBBR) may occupy the CutC active pocket. 2. Microbiota Modulation: Downregulates cutC/*cntA* gene abundance; alters community structure. 3. Multi-target Regulation: Also modulates host hepatic FMO3 activity.	Strong (Multi-level & Clinical) • Clinical efficacy: Reduced plaque score in AS patients. • In vivo efficacy (Animal): Reduced plasma TMAO & atherosclerotic plaques. • Microbiota remodeling & gene downregulation. • Direct inhibition via dhBBR.	In vitro: Inhibits TMA production in human fecal microbiota cultures. In vivo (*ApoE*^−/−^ mice): Significantly lowers plasma TMAO; attenuates choline diet-induced atherosclerosis. Clinical: Reducing plasma TMAO leads to a 3.2% reduction in plaques	Very low oral bioavailability; relies on local gut action; may cause GI discomfort.	[54,72]
Resveratrol (Stilbenes)	1. Indirect: Remodels gut microbiota (inhibiting TMA-producing bacteria); increases bile salt hydrolase (BSH) activity. 2. Potential direct inhibition (docking prediction)	Moderate • In vivo efficacy (Animal): Reduced TMAO & atherosclerosis. • Microbiota remodeling. • In vitro activity: Inhibits TMA production in cecal content. • Resveratroloside had the highest Vina score.	In vitro: Reduces TMA yield from gut microbiota cultures. In vivo (*ApoE*^−/−^ mice): Attenuates TMAO-induced atherosclerosis; associated with increased beneficial bacteria.	Low bioavailability; efficacy is highly dependent on the presence of gut microbiota.	[73,74]
PMFs (Polymethoxylated Flavones)	Potential Direct Inhibition: Molecular docking suggests binding to CutC active site; directly inhibits *cntA/B* and *cutC/D* enzyme activity in vitro.	Moderate (Biochemical) • In vitro direct enzymatic inhibition. • Molecular docking prediction. • Cellular efficacy (HepG2): Down-regulates FMO3 mRNA, reduces TMAO formation.	In vitro: Significantly inhibits *cntA/B* and *cutC/D* enzyme activity; reduces TMA generation in microbial assays. Cellular (HepG2): Reduces TMAO formation in TMA-induced cells.	Lacks in vivo atherosclerosis model data. Mechanism and potency need strict validation.	[75]
Tea-derived Flavonoids	Potential Direct Inhibition (Predicted only): Molecular docking suggests binding to TMA-lyase (CutC).	Preliminary • Molecular docking prediction only (e.g., Kaempferol 3-O-rutinoside had the highest Vina score).	In silico: Docking studies indicate potential interaction with CutC active site.	Evidence is entirely computational. Requires biochemical and biological validation.	[76]

**Table 3:** Summary of clinical and preclinical evidence for reducing TMAO levels.

Intervention Type	Intervention	Study Design	Population/ Sample Size	TMAO Changes	Key Conclusions/ Mechanisms	References
Dietary Intervention	Mediterranean Diet (Red Meat Restriction)	RCT Feeding Trial	Overweight/ Obese Adults (*n* = 39)	Significantly Decreased (3.1 vs. 5.0 μM)	Restricting red meat intake (200 g/w vs. 500 g/w) can effectively reduce fasting serum TMAO.	[77]
	Short-term Mediterranean Diet	Randomized Crossover Trial	Healthy Volunteers (*n* = 20)	Significantly Decreased	Short-term intervention can reduce plasma TMAO, supporting its cardiovascular protective effect.	[78]
	Dietary fiber	Randomized Crossover Double-blind	Healthy Volunteers (*n* = 13)	Subgroup effective	No overall difference, but can attenuate TMAO elevation after a beef meal in low-meat consumers, *cutC*.	[79]
	Inulin	RCT	T2DM high-risk population (*n* = 18)	No significant change	No decrease in fasting/postprandial TMAO after 6 weeks (negative result).	[80]
Microecological Intervention	Probiotics	Double-blind RCT	Healthy Males (*n* = 40)	Trend of decrease	Did not significantly reduce postprandial TMAO AUC, but the proportion showing a downward trend was higher than the control group.	[81]
	Synbiotics	Double-blind RCT	Patients with Dyslipidemia (*n* = 56)	Significantly Decreased	12-week intervention significantly reduced serum TMAO and endotoxin.	[82]
Natural Products	Berberine	RCT	Patients with Hyperlipidemia and AS (*n* = 21)	Decreased by 35% (blood) 29% (feces)	Directly inhibits CutC/CutD enzyme activity and improves arterial plaques.	[54]
	Berberine	Intervention Study	Hypertensive Patients (*n* = 15)	Decreased by 8.8–16.7%	Binds to and inhibits CutC enzyme activity, inhibiting the biosynthesis of TMAO precursors in the gut microbiota.	[83]

## Data Availability

No new data were created or analyzed in this study. Data sharing is not applicable to this article.
